# *CHATogether*: a novel digital program to promote Asian American Pacific Islander mental health in response to the COVID-19 pandemic

**DOI:** 10.1186/s13034-022-00508-4

**Published:** 2022-09-23

**Authors:** Jae Eun Song, Nealie T. Ngo, Jessica G. Vigneron, Alan Lee, Steve Sust, Andrés Martin, Eunice Y. Yuen

**Affiliations:** 1 Department of Comparative medicine, Yale School of Medicine, New Haven, CT 06511 USA; 2 University of Toledo College of Medicine and Life Sciences, Toledo, OH USA; 3 Mailman School of Public Health, Columbia University, New York City, NY USA; 4 University of British Columbia, Vancouver, BC Canada; 5 Stanford University School of Medicine, Palo Alto, CA USA; 6 Yale Child Study Center, New Haven, CT USA; 7 Department of Psychiatry, Yale School of Medicine, 300 George Street, New Haven, CT 06511 USA

**Keywords:** Virtually delivered intervention, Culturally-informed mental health, Asian American and Pacific Islander (AAPI), Qualitative methods, and COVID-19

## Abstract

**Background:**

In response to the COVID-19 pandemic and the associated rise in anti-Asian hate crimes, we developed the Compassionate Home, Action Together program, (*CHATogether*) to support the mental health of the Asian American and Pacific Islander (AAPI) community. *CHATogether* is a culturally informed and virtually delivered support program that harnesses the talents of AAPI teens, young adults, parents, and mental health professionals who share a commitment to serve their local communities.

**Methods:**

Our objective was to identify the active components, optimal utilization, potential benefits, and pertinent limitations of the *CHATogether* program during the 3 years since its inception in 2019. By that time, the program had developed six distinct component arms: interactive theater, mental health education, research, peer support and community outreach, collaboration, and AAPI mentorship. To work towards this objective, we conducted a qualitative study using thematic analysis and an inductive approach based on grounded theory (GT), in which we analyzed anonymized transcripts of four focus groups, comprised of 20 program participants (11 females; 9 males).

**Results:**

We developed a model of two overarching domains, each with three underlying themes: I. Individual stressors: (1) Family conflict; (2) Cultural identity; and (3) Pandemic impact; and II. Collective stressors: (1) Stigma related to mental health and illness; (2) Pandemic uncertainty; and (3) Xenophobia and societal polarization. Strengths of the *CHATogether* program include its role as a conduit toward AAPI connectedness and pride as well as purpose in building community. Through support and mentorship, the program cultivates a unique platform that promotes healing and resiliency in response to pandemic stressors and beyond.

**Conclusions:**

*CHATogether* creates a safe space for the AAPI community. Through its methods of storytelling and encouraging creativity, *CHATogether* facilitates the discussion of challenging topics specific to the AAPI community. Given the national mental health crisis that is further being exacerbated by the COVID-19 pandemic, a digital prevention program such as *CHATogether* holds promise towards providing access to mental health resources and supporting early help-seeking behaviors for individuals in the AAPI community.

## Introduction

The coronavirus disease (COVID-19) has been labeled as a “Once-in-a-Century Pandemic” [1] that has upended lives everywhere. The global increase in stress, anxiety, depression, and post-traumatic stress disorder (PTSD) [2–4] due to COVID-19 are apparent in society and well documented in the medical literature. Stressors stemming from the pandemic such as prolonged confinement, “cabin fever,” unemployment, and loss of social resources, have all contributed to an increased risk for suicide, parental alcohol abuse, and domestic, child, and sexual abuse [5–7]. Furthermore, COVID-19 has tested the boundaries of even well-functioning families, leading to an increase in impatience, annoyance, and hostility within homes [7, 8].

Propaganda, sensationalism, and misinformation about COVID-19—what the World Health Organization (WHO) calls an infodemic [9]—has also incited mass panic, fear, and distrust among communities. It has been said that the panic stoked by “fake news” spread faster than the coronavirus itself [10, 11]. As political leaders disseminated ominous messaging and misconceptions about COVID-19, such as calling the disease “The China Virus,” already devastated AAPI communities became the victims of xenophobic attacks. Individuals reported being spit on, yelled at, and physically attacked, with victims ranging from children, women, and the elderly. Among Chinese American families, 32% of parents and 46% of youths reported COVID-19 racial discrimination online, with this number reaching over 50% in person [12]. *Stop AAPI Hate*, a non-profit organization, which tracks incidents of discrimination against AAPIs, reported more than 10,000 hate incidents between March 2020 to December 2021 [13]. The rise in xenophobia and anti-Asian sentiment may worsen AAPI family harmony and create emotional disconnection in the home [14].

Compassionate Home, Action Together (*CHATogether*) is a digital wellness program tailored specifically for Asian American and Pacific Islander (AAPI) teens, young adults, and families. The program was developed as a response to the stressors experienced within AAPI communities as a result of COVID-19. The program started as an in-person workshop in January 2020, when many Chinese international students in the United States (U.S.) worried about the health of their families back home as COVID-19 spread in Wuhan, China. The fledgling peer support group eventually became a critically needed safe space for individuals to openly discuss their wellbeing, with its overall message of, “Don’t let fear be contagious.” As COVID-19 began shutting down communities, *CHATogether* revamped its program to become solely digital, offering AAPI peer support through social media and virtual programs. Live theater webinars provided cultural-informed prevention strategies to tackle issues such as AAPI hate crimes and discrimination as well as how to process and show solidarity after tragedies such as Atlanta’s mass shooting targeting Asian women in March 2020. *CHATogether*’s virtual space has also given AAPI youth the opportunity to cultivate their cultural pride and showcase their own projects with the *CHATogether* mentorship. As detailed in the Methods section, *CHATogether* has been uniquely built to handle complicated AAPI family conflicts, help bridge the acculturative gap between parents and children, and break down the stigma of difficult conversations surrounding topics such as racial bullying, suicide, and gender dysphoria [15, 16].

The COVID-19 pandemic has skyrocketed our worries about health and safety, as well as forced our nation to reflect about deep-seated issues pertaining to racism and bigotry. The pandemic further exacerbated underlying xenophobia and the stigma around mental health, illnesses, and treatment for AAPI individuals. As members of the AAPI community have already been among the least likely to access mental health services [17], this issue widens an already dire gap. We developed *CHATogether* in an effort to break the silence against racism and the growing mental health crisis affecting adolescents, young adults, and families in the AAPI community. The program includes six different creative modalities intended to address cross-cultural and cross-generational needs, as well as to promote mental health and wellbeing. In this article we describe the development and operationalization of *CHATogether* and present findings from a qualitative study based on the experience of the program during the 3 years since its inception.

## Methods

### *CHATogether*: program development

#### Theoretical context

*CHATogether* is consistent with the mental health awareness and prevention goals of Strategic Prevention Framework (SPF) from the U.S. Substance Abuse and Mental Health Services Administration (SAMHSA). The SPF emphasizes community needs, building, and implementation of evidence-based prevention strategies through grassroots coalition efforts. Moreover, SPF incorporates elements of cultural humility [18] and communities’ values, traditions, distinctive heritage, and social structures [19]. SPF has been applied in several adolescent mental health initiatives [20, 21]. Drawing from the SPF, *CHATogether* uses approaches centered around the AAPI community needs to promote mental health prevention during the COVID-19 pandemic [19]. The program exemplifies Participatory Action Research (PAR) [22–24], in which the intended beneficiaries are part of the research effort. PAR in turn incorporates *cooperativism*, a modified economy model where the program is composed of individuals who are part of a collective member-based ownership [25]. In this cooperative alliance approach, youth and adult volunteers partner based on shared values, goals, and passions to bring positive influences to their communities at a time of crisis [25]. In *CHATogether*, members draw on their strengths, talents, and interests and use the guiding principle of altruism to help address AAPI mental health and anti-Asian sentiment.

The interactive theater component of the program incorporated elements from the “Theater of Oppressed (TOp)”, which was first developed in Brazil in the 1970s by dramatist Augusto Boal, influenced by the work of educator Paulo Freire and his classic Pedagogy of the Oppressed (1970) [26]. TOp aims to promote social changes in the community by promoting non-hierarchical dialogue between audience and performer [27, 28]. TOp has informed medical education programs [29], public health initiatives on sexual assault [30], and cancer screening awareness [31]. We further adapted its tenets to the AAPI community’s needs during the pandemic.

#### The program’s six components

**Fig. 1:**
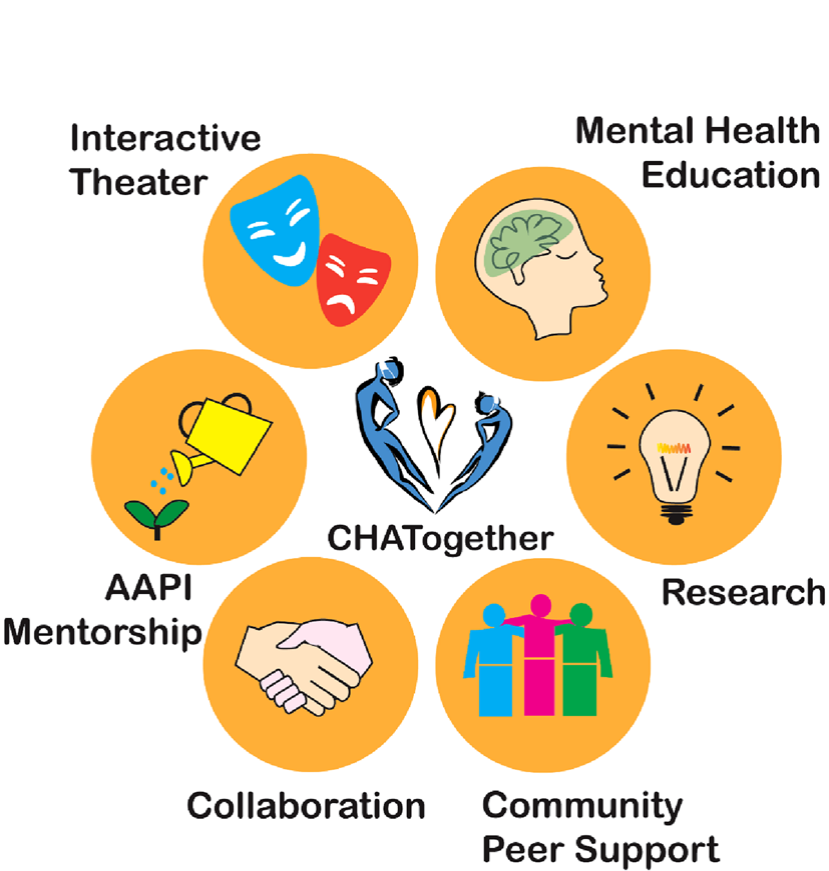
Schematic framework of *CHATogether*’s six core components. Artwork by Dr. Chiun Yu Hsu

**Fig. 2:**
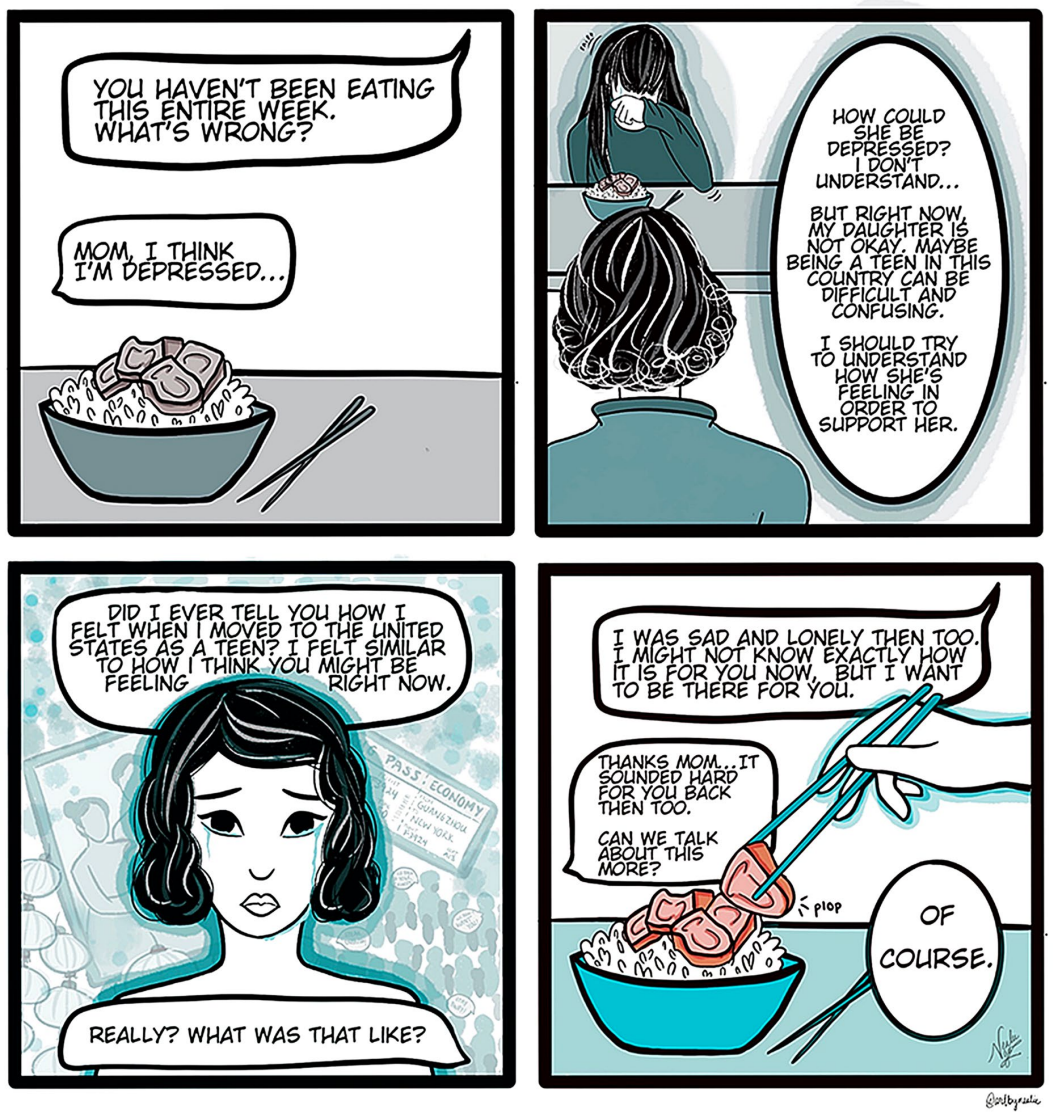
Example of mentalization in an AAPI context during a mother–daughter interaction. Illustrative panel from the graphic novel, “Mentalization: A Comic About Asian American Mental Health”. Artwork by Nealie T. Ngo, MPH

**Fig. 3:**
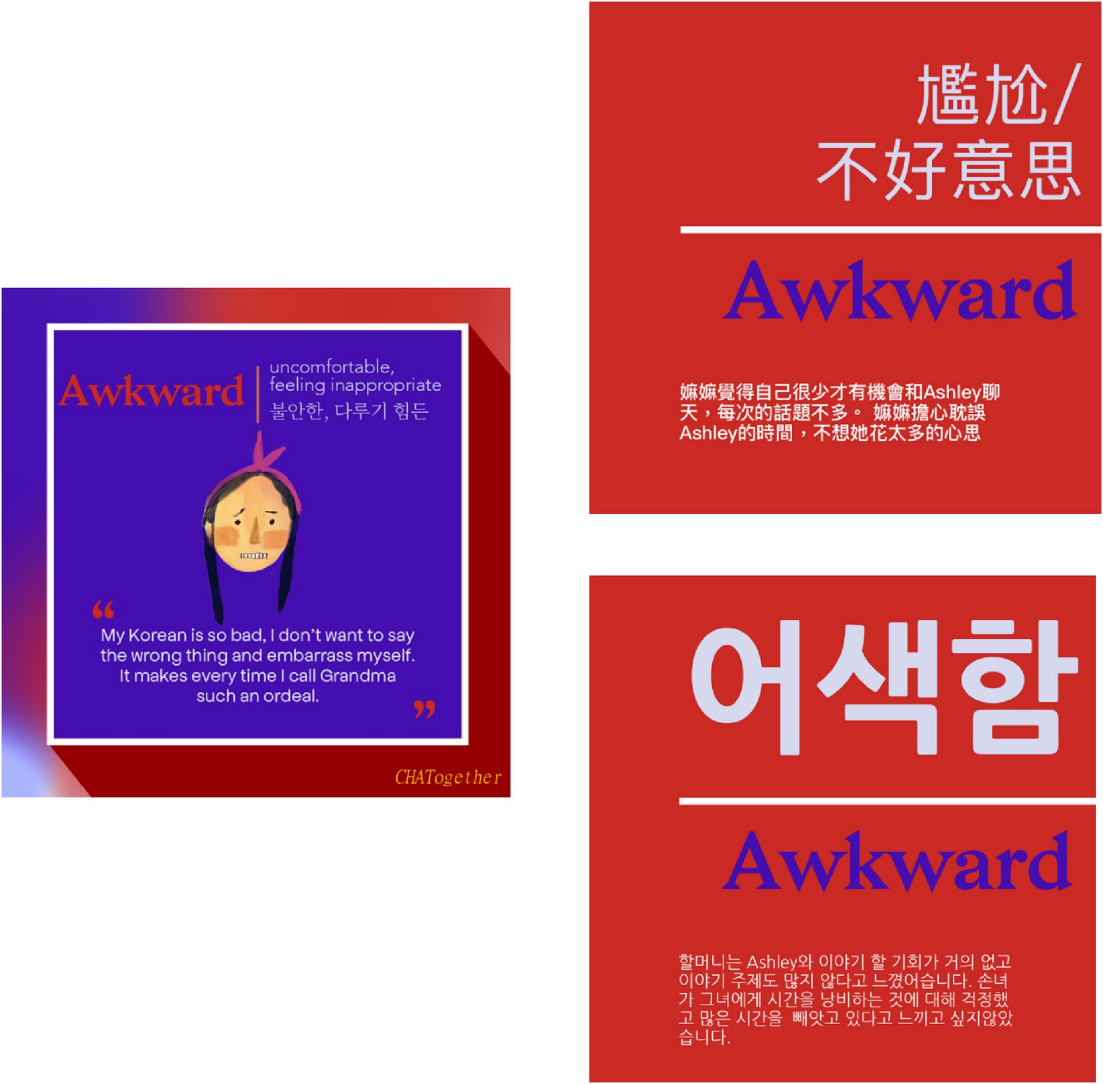
Representative flashcards for “awkward feelings”. Flashcards based on the *CHATogether* skit “*Grandparent–grandchild language barriers and overcoming them*.” To help bilingual parents and grandparents understand various emotional expressions, flashcards are translated into multiple Asian languages, including Chinese and Korean (upper and lower panels on the right, respectively). Artwork and translation by Joan Yang and Violet Tan

*CHATogether* harnesses the potential of storytelling and the arts, leveraging electronic platforms to address the dynamics of family life and to translate the stigmatized language of mental health into stories told through theater, visual arts, and other expressive outlets. *CHATogether* has six core components: (1) interactive theater; (2) mental health education; (3) research; (4) community peer support; (5) collaboration; and (6) AAPI mentorship (Fig. 1,2, 3 and Table 1). Each component is led by leaders from a pool of 30 to 40 standing members, each of whom contribute their expertise in areas of improv, acting, graphic medicine, visual arts, research, psychotherapy, and community outreach. At a time of strict COVID-19 restriction, including lockdown and shelter-in-place mandates, *CHATogether* was launched as an interactive, theater-based mental health program disseminated through dedicated social media channels. Members meet biweekly to share project updates and peer supports, as well as gathering and milestone celebrations.

**Table 1:** Six core components of the *CHATogether* initiative

Component	Description and active elements
Interactive theater	• *CHATogether* invites members to translate their own child-parent scenarios of conflict into interactive dialogues • Working with mental health clinicians, we created videos on topics such as stigma, emotional management, and various cross-cultural and mental health challenges common among AAPI child-parent dyads. We also created videos relevant to the COVID-19 pandemic, including depression and anxiety in the context of anti-Asian sentiment and hate crimes, substance use in teens during COVID-19, and stressors specific to the AAPI LGBTQ community • Developing and watching dramatized skits invites reflection and dialogue to “make visible the invisible” and fosters transformative processes • Videos are shared for public access through social media channels, such as *Youtube, Facebook, Instagram, and Tiktok* • *CHATogether* has created 26 theater skit videos in the first three years since its inception. Representative videos: • https://www.youtube.com/watch?v=eTl3BcEK41s&t=2s • https://www.youtube.com/watch?v=UEyXQ-TnrBE
Mental health education	• Through three different media platforms (bilingual flash cards, a graphic novel, and a podcast), we summarized key points embedded in the theater skits, such as social emotional learning and techniques for improved parent–child communication • Figure 2 is a representative panel from a graphic novel demonstrating the concept of mentalization during an AAPI mother–daughter interaction • Communication challenges in AAPI families may include language barriers and cross-cultural differences in emotion expression [32]. To address these unique needs, we developed “feeling flashcards” describing emotions expressed during the theater skits (as exemplified in Fig. 3) • To increase access for bilingual parents and grandparents, we translated all educational materials into several Asian languages including Chinese (Simplified and Traditional), Korean, and Vietnamese
Research	• We explored the program’s active components through a qualitative approach • Team members and participant-beneficiaries led the research design, data collection, analysis, and presented results at academic conferences during the first three years since the program’s inception (n = 29; e.g., “*Bridging the Cultural Divide: The Creative Use of Digital Media to Engage Adolescents and Their Families Around Mental Health*” was presented at the 69^th^ American Academy of Child and Adolescent Psychiatry Annual Meeting
Community peer support	• Since 2019, we have held a total of 38 *CHATogether* community events, collaborated with academic institutions, high schools, community-based organizations, and churches (17 local; 17 national, across 10 states; and 4 international, across 3 countries) • Conference attendees spanned between 30 and 200 participants, including adolescents, transitional age youth, and parents from the host AAPI community • In some cases, we invited panel discussants, including mental health providers, medical students/trainees, school educators, and AAPI community leaders
Collaboration	• Given the cross-disciplinary approach of *CHATogether* with the creative arts, we collaborated with community artists, theater members, and filmmakers in the AAPI community to promote mental health • *CHATogether* clinicians and artists connected through a serendipitous process fueled by word of mouth, mutual solicited interests, and post-event outreach for collaboration • Artists’ creativity synergized with clinicians’ professional knowledge, contributing to the novel intervention. Specifically, creative art delivers a culturally informed content to the AAPI community
Mentorship	• The dynamic nature of program members spans multiple developmental ages and training levels. When collaborating in a team, junior members benefit from mentorship on career and personal guidance from senior members • Members form workgroups of 3 to 5 to conduct specific activities. Each workgroup is led by an individual in a more advanced career stage such as a psychiatry faculty or trainee. Other workgroup members are more junior in their careers, such as nursing or medical students, undergraduate or high school students • All workgroup members share a common interest in children’s mental health • AAPI mentorship additionally provides support for situations in which members experience anti-Asian sentiment in their communities

#### Interactive theater

A moderator co-constructs a skit with community participants, in an effort to address a problem in their daily lives. The moderator next engages the audience using “time outs” and “time ins” to “freeze” and “replay” the skit at various points, until a realistic solution to the problem is found [28]. Similar interactive theater models have been used in medical education [29, 33], *CHATogether*’s interactive theater skit includes three sequential parts:

##### Improvisation

*CHATogether* invites members to construct a role play dialogue based on their own parent–child conflict scenarios. Two audience members, each improvising as a teen and a parent, are guided by a professional with expertise in improvisation and character development. Next, all members involved in the improvisation process discuss further enhancements toward cultural adaptation, relatability, and extraction of potential teaching points.

##### Video production

We recorded interactions among consenting participants (n = 39). Each video was facilitated by an AAPI child and adolescent psychiatrist. In each of the 26 videos: (1) participants act out the problematic scenario; (2) the clinician-moderator helps discuss a process towards mentalization of each other’s perspectives [34, 35]; and (3) the participant-actors then re-play the same scenario, this time incorporating more effective communication skills leading to an alternative and better scenario and outcome. We recorded all videos using synchronized videoconferencing via Zoom (San Jose, CA).

##### Video dissemination

After editing, videos were made available to the public on social media through dedicated social media channels: https://www.youtube.com/channel/UCRX2Nzv65ekzHikAaiyG6YQ/videos; https://www.facebook.com/CHATogetherWithUs

### Qualitative study of the program’s rollout

#### Participants

Twenty individuals (18–30 years old) participated in four different focus groups, each with members with differing levels of seniority and involvement in *CHATogether*. We summarized participant demographic characteristics in “Appendix”.

#### Procedures

We conducted the focus groups in the fall of 2019 and spring of 2020 using synchronous videoconferencing. We obtained institutional review board approval from the Yale Human Investigations Committee (Protocol # 2000028490). At the beginning of each session, participants provided verbal consent for audio recording. We discarded video content and had audio files transcribed by Rev.com (San Francisco, CA and Austin, TX). We de-identified transcripts before analyzing them supported by NVivo 12 software (QSR International, Melbourne, Australia).

The senior author (EC) led four focus group interviews to discuss different aspects of *CHATogether* and/or COVID-19. Participants in focus group A (“*CHATogether* as a peer support group during the pandemic”) were asked to describe their relationship with *CHATogether*, how they first got involved with the group, what (if any) benefits *CHATogether* had provided (such as a sense of purpose, stability, flexibility, a safe space, and community), thoughts on *CHATogether*’s ability to provide opportunities for artistic outlets, and how COVID-19 had affected them. Focus group A was mainly conducted with the founding members, as their involvement in the program allowed a deeper reflection on *CHATogether*’s mission, foundation, implementation, and vision. *CHATogether* was founded during the peak of the COVID-19 pandemic when anti-Asian hate became a significant concern in the community. Initial codes suggested several significant stressors within the family and community, and thus we expanded those themes through the subsequent focus groups. Participants in focus groups B and C, “Effect of COVID-19 on family harmony” [14], consisted of a mix of founding members, newly joined members, and non-members. We asked them to describe their family dynamics before and during the pandemic, any cultural or communication gaps that occurred and/or were heightened during that time, and whether any coping skills were learned as a result of involvement with *CHATogether*. In case of non-members, an introduction of *CHATogether* work (such as skit videos and webinars) was shown before the interview. Focus group D was an expansion of preliminary themes from previous interviews that many participants identified. These themes included racism as a significant stressor during the pandemic. Participants in focus group D (“Racism and stress on AAPI individuals”) were asked to describe their thoughts and experiences with anti-AAPI discrimination before and during COVID-19, the psychological impact of racism at the individual, family and community levels, different ways to communicate about racism and cultural identity with family, and any other support networks.

#### Data analysis

For this qualitative project we used thematic analysis in an inductive fashion [36], which was rooted in an interpretative phenomenological approach aimed at examining the participants’ personal and subjective experiences [37–39]. Three authors first worked independently to identify and organize codes. Those raw codes were then shared with the other investigators to triangulate a final codebook. We created a list of overarching domains and underlying themes based on the codebook, each supported by several verbatim quotes. We analyzed data iteratively until reaching theoretical sufficiency [40]. We adhered to accepted guidelines for qualitative research [41, 42].

## Results

We developed a model of two overarching domains, each with underlying themes and subthemes: Domain I: stressors experienced by participants; Themes: (1) Individual; and Subthemes: (1a) Family conflict; (1b) Cultural identity; and (1c) Pandemic impact. Collective: (2a) Stigma related to mental health and illness; (2b) Pandemic uncertainty; and (2c) Xenophobia and societal polarization. Domain II: Benefits to participants: (1) Individual: (1a) Expression of creativity and talent; (1b) Productivity and sense of purpose; (1c) Connection; (1d) Mentorship; and (2) Collective: (2a) Consistency; (2b) Sense-making through storytelling; (2c) AAPI support group; and (2d) Provision of a safe space for stigmatized conversations.

## Domain I: stressors experienced by participants

### Individual stressors

#### Family conflict

Many participants identified family conflict as a general stressor. Communication barriers became significant sources of child-parent conflict. Many participants perceived their families as lacking emotional engagement to each other’s feelings and suffering. One participant stated how dismissed they felt, given that:

“Whenever I talk to them about anything that I'm concerned about with my feelings, or if I feel like I don't fit in socially, or challenges that I've had with me and my racial identity, social identity, they just say, ‘Oh, okay.’

Several participants attempted to embrace family harmony by avoiding conflictual communications. One participant stated how:

When I was younger, I thought they [my parents] understood. Now, I realize that they just don't, and they don't want to tell me why. I realize that I do that with them as well.

Moreover, many participants live in a household with older generation or first-generation immigrant family members. They identified that language and generational barriers also forestalled complicated conversations, as well as more intimate conversations requiring emotional support.

Whenever they try to talk to me in Chinese and there's words I don't understand, I find it very difficult to be patient to try to use other words to explain to them.

#### Cultural identity

Several participants noted their families’ experiences with cross-cultural challenges across generations. Differences in cultural expectations in a child-parent relationship at times hindered the development of healthy family dynamics among immigrant families. While acknowledging the broad diversity of Asian culture, Confucianism is a common historical influence on the many Chinese descendants living in countries across most of East Asia and, to a lesser extent, of Southeast Asia, a fact relevant to most study participants. Confucianism focuses on moral values such as filial piety, children’s primary duty of respect, obedience, caring for parents and elderly members of the family [43]. Such concepts highlight some unique aspects of Asian heritage, including hierarchical relationships, a more authoritarian parenting style, and emotionality [32]. Parents may perceive speaking about emotions or personal problems as disrupting family harmony, whereas for children the opposite may be the case. As a result, conflicts among family members were commonly ignored rather than discussed openly. Such discrepancy of cultural values, expectations, and family functioning commonly prevented children from communicating about their struggles when they needed family support the most.

Some participants reported cultural and generational tensions in the understanding of family harmony between parents who grew up in the mainland and their American-raised children: “If a child decides to break the values that a family has set, that is seen as betrayal.” Straying from the family’s traditional values and predefined pathways were seen as a disharmonious affront to their parents’ viewpoint.

Many participants expressed confusion or avoidance of their cultural identity as both Asians and Americans. They noted that first-generation immigrant parents tended to prioritize assimilation and success in the US and impose high expectations on their children, over the role of emotional validation, overall wellbeing, and racial identity development. Some felt pressured to assimilate into the dominant white culture, a process that made them feel ashamed of their Asian heritage:

When I was younger, I claimed myself as an American. I would get into arguments with my grandmother where she would say I was Chinese, but I opposed that: ‘No, I'm American.’ I would refuse to use chopsticks because I would say only Chinese people use chopsticks. I'm not Chinese so I can't use them (Table 2).

**Table 2:** Domain I: stressors experienced by participants

Theme	Subtheme	Representative quote(s)
1. Individual stressors	*Family conflict*	My family harmony? It feels like we pretend not to have a conflict. No one wants to talk. And avoidance, I presume is doing something good for each other. But we have zero communication, in our so-called “harmony.” There is a sense of ownership that my parents had. “I feel like I'm not a human to them. I feel like I'm an object that they control.” I feel like they see me as something they have ownership of and that doesn't have their own conscience.”
	*Cultural identity*	When I was younger, I would only say that I was American. To be frank, I didn't want to tell people that I was Chinese. You want to fit in, you don't understand why you're different, and you hate that you are different, but I think part of growing up is accepting the things that make you different and embracing them and finding places that are special
	*COVID-19 pandemic*	Being in the same physical space day after day causes extreme conflicts COVID, sort of united the family: feeling the same, sharing similar feeling, and feeling safe to express them
2. Collective stressors	*Mental health stigma*	The shame and guilt of talking about mental health as a weakness. It's like, “Oh yeah, I have this pain, I have this sneezing. I have the fever. It is like more objective neutral thing to talk. But mental health, that's so stigmatized to talk about that.”
	*Uncertainty from COVID*	In COVID-19, we are like floating on the ocean, we have no idea what is coming next. And things keep piling up Things that I see on the news make me feel that I am uncertain about what my future holds: “Where should I move to build my family and find the place where I belong?”
	*Polarized Society and family conflicts*	Issues around injustice, inequality, and racism have been ongoing. [My family and I] are not necessarily in active conflicts, just disagreements, not seeing eye to eye on these issues, and not really talking about them either, because of not much understanding on either side. Instead of bringing up frustration, which ends up being something that I'm okay with, but I think my mom is not. For example. She would rather spend our time to share things that pertain to our individual lives rather than to the broader society

#### COVID-19 pandemic

The pandemic exposed and amplified individual problems and pre-existing conflicts: “Being in the same physical space day after day causes extreme conflict.” Identified stressors included financial difficulties and job loss or insecurity. The instability and anxiety from financial difficulties exacerbated family problems, especially during the protracted quarantine and periods of mandated lockdown. Unemployment with no colleagues to interact with and with no physical workplace exacerbated tensions between family members:

I also felt very grateful that I had a job and I have a roof over my head, but also a lot of sadness and anxiety of like, “Oh, what will happen to me? Will I also be in a similar situation [as the less fortunate]?

Despite a lack of personal space, which led to heightened conflicts, family members were also forced to be more emotionally communicative and united in facing the unprecedented challenges imposed by the pandemic. One participant explained how:

COVID-19 has brought us closer together and being a little bit more emotionally expressive. Not quite 100% there yet, but I think that forced my dad to be a little bit more open.

### Collective stressors

#### Mental health stigma

Participants largely agreed that mental health in Asian families and communities is a stigmatized subject of conversation. Perception of weakness and lack of supportive spaces associated with shame and guilt of having mental health struggles were discussed as significant stressors. As one participant explained, “Specifically in Asian immigrant communities, mental health is viewed in a very negative way, like ‘What's wrong with you?’” Another participant highlighted the discrepancy in openness compared to their peers at school:

I know for a lot of my friends at school, they're able to talk with their parents about what's wrong and how their parents can help. But in Asian communities, talking about mental health is so taboo.

#### Uncertainty from COVID-19

The COVID-19 pandemic led to a massive disruption in longstanding social structures and routines, forcing people to adjust to new environments while simultaneously disbanding others. As one participant explained:

It was such an uncertain time and there was so little that any individual could do, and so much of what we were all being asked to do was to not do anything.

Many participants identified the division and polarization of society, especially reflected in the increase of racial violence against Blacks and Asians, as another aspect of pandemic uncertainty. They expressed fear, hopelessness, helplessness, and a lack of control. One participant stated, “Having COVID-19 occur with *Black Lives Matter* and social movements where we're once again in a situation that’s very uncertain, the feeling of wanting to help and wanting to be able to do something but not being able to do much.” Another participant shared her traumatic experience on “being called Coronavirus” by a fellow high school student. Yet another participant commented how “the murders in Atlanta were a kind of climactic moment where it triggered all these feelings from years, decades of built-up micro-aggressions or macro-aggressions. It was certainly an awakening”. A few other participants experienced concerns over their personal and family safety:

Am I going to get jumped by someone for being who I am?

#### Polarized society and family conflicts

Contributing to their feelings of anxiety and hopelessness, several participants identified how societal uncertainty further intensified their family conflicts and emotional tension. They expressed how family members could not provide support or agreement due to divergent political opinions, cultural values, and language barriers:

When talking about social issues, I have not even tried to bring that conversation up to my parents, because of what they've been going through, and because we're so far away. Also, my lack of ability to speak in their language, even though they are proficient in English. They don't encounter terms like ‘model minority’, or ‘civic engagement’.

In face of anti-Asian racism, participants expressed frustration to parents’ indifference of the discussion, “My mom came here for graduate school, and on paper being successful, it’s frustrating to me that anti-Asian racism is not a big of a deal to her.”

## Domain II: *CHATogether*’s benefits to participants

### Individual benefits

#### Expression of creativity and talent

The personal benefits of unleashing and making use of creativity and talent was identified among several participants. *CHATogether*’s projects provided a platform to share thoughts and feelings through art and less traditional means of communication. One participant stated how:

We translate languages into action, into word, into video, into something that can be understood by everyone. The conversation becomes more tolerable and acceptable to talk and to express.

Creative expressions enabled stigmatized topics like mental health and racial trauma to become more approachable, intimate, and descriptive. It also provided a healthy coping outlet for developers who were involved in the different component projects. As described by one of them:

It carries incredible value to see effective communication demonstrated as an educational model. It also allows us to interpret our own experiences in possible alternative ways in which they could occur.

#### Productivity and sense of purpose

Participation in *CHATogether* created a sense of action and control, which was especially important during the pandemic and its lingering—and ongoing—aftermath. As one participant shared:

Purpose is the main fuel and anchor for productivity.

Many members identified altruism and helping others going through similar struggles as the purpose of this group. Sense of purpose also strengthened participants’ sense of control. Making something out of challenging times taught adaptability, as described by one participant: “It also speaks to the flexibility, the cognitive flexibility, that helps us think outside the box…it can be the silver lining of adversity.” It was also helpful in overcoming the struggles in the ongoing pandemic:

As opposed to the powerlessness and sense of guilt, we expanded *CHATogether* during COVID. I feel like it gives me the power to put my mind into action as we contribute to something or someone out there (Table 3).

**Table 3:** Domain II: *CHATogether*’s benefits to participants

Theme	Subtheme	Representative quote(s)
1. Individual benefits	*Expression of creativity and talent*	The power of the arts can really transcend a level of conversation where you can connect with others and really be able to convey a sense of unity, compassion, care, and empathy with others. I think it can do so in a way that other modes of communication cannot
	*Productivity and sense of purpose*	Here we take the experiences that we have and channel them into a certain type of product. Because we do that, which requires a certain level of empathy, mentalization, of thinking around yourself—outside of your own box—for the purposes of helping others. That, combined with the fact that so much of the work we do and the way we do our work has changed with the pandemic, and that the topics we're discussing are related to the pandemic. It's been a really nice way for me to bring reason to what's going on We are not only are united as a group, but also bring it forward to other people, to the community, in a way that we hope can be helpful
	*Connection*	It's a sense of loss and confusion. You don't know what the next moment is going to be; society adding on more and more, but we have something important to hang on to: we have this group, the cohesiveness of being together
	*Mentorship*	The different career paths of my senior colleagues affirm that no matter what I end up doing, it is always possible to make time and invest effort for issues we care about. As a young person beginning to venture into the real world, *CHATogether* is the perfect place for me to hone instrumental life skills like collaboration and speaking up, and it makes me excited for the kind of research and initiatives I hope to pursue
2. Collective benefits	*Consistency*	“The structure, and the consistency, and the meaning for us to work in *CHATogether* gives us a lot of hope against what the COVID-19 pandemic is putting upon us.”
	*Sense-making through storytelling*	“I know so many other people's stories that have shared themes of some of the things that *CHATogether* hopes to address— or at least even begin to explore, to bring light to some discussions. These conversations allow for new narratives to be created. Healthy narratives that can show that it's possible when things within a family or within an individual feel impossible, when one feels there's no model or no way out. in these situations.”
	*AAPI support group*	“I think that *CHATogether* has a very powerful potential to connect AAPI for those who are maybe at different levels of accepting or appreciating their [Asian] heritage. Share their experiences and understand that a lot of things that you feel like and are maybe you're going through on your own are actually quite common, and maybe that brings on a belief and connection to others.”
	*Provision of a safe space for stigmatized conversations*	The skit allows a medium so that we don't need to talk directly at each other but instead we're talking within the skit or through other artistic outlets. It is a more tolerable, less taboo medium, in which we can discuss things in a culturally sensitive way. I hope that we can expand to many other minorities, not just AAPI. *CHATogether* has so much potential to bring people together, to let them feel comfortable to talk about mental health.”

#### Connection

The importance of interpersonal connection and teamwork was shared by all participants. These relationships were particularly critical to people who experienced limited or highly suppressed social interactions in their family or community: “Sharing the experience of struggle with somebody who gets it and then laugh about it at the same time is a form of healing.”

#### Mentorship

*CHATogether* members include a wide range of passionate individuals in various career developmental stages and disciplines, including the arts, theater, medicine, public health, science, and architecture. Connection through collaborative projects create additional mentorship components in the program. Senior *CHATogether* members represent a symbol of “cultural pride” for junior members to model after. One participant stated how:

Working with AAPI senior colleagues has shown me what is possible in terms of career paths, possible academic areas to which I can contribute, and AAPI collaboration. The *CHATogether* environment exemplifies AAPI people supporting one another in the confusing political environment that erases and sometimes destroys our identities.

### Collective benefits

#### Consistency

*CHATogether* successfully adapted to digital programming during the pandemic, which provided structure and consistency. Members met bi-weekly, led by the program director. Each meeting started with bond-building conversations about timely concerns in the AAPI community, followed by a list of project items on the agenda to discuss progress and future directions. *CHATogether* also held team workgroups, social events, and holiday and programmatic milestone celebrations between regular meetings: “We are coming from different places in the U.S. and other places. Since the difficult time of pandemic, we are still consistently meeting regularly. We are happy to be doing this meaningful work. We grow so much, are proud of each other, and stay emotionally healthy together.”

#### Sense-making through storytelling

Another community benefit that was identified was the power of storytelling in making sense of overwhelming, confusing, strange, and unprecedented times. Sharing personal stories creates a strong bond within the community, especially when sharing relatable experiences:

Being able to hear from other Asian Americans is healing. These are issues that I’ve thought about and struggled with; how you communicate. I wish we were able to talk about them more.

Storytelling allows both tellers and listeners to process the experience from various perspectives through empathy, exchange, and reflection. Sharing stories seemed to allow for sharing a considerable amount of emotional burden. One participant stated how, “Each story is a personal narrative. In the process of sharing it, we're trying to put our mind and feelings into words and actions.”

#### AAPI support group

*CHATogether* successfully created a collaborative environment that serves as a support group for its members through creative and productive outlets. The cooperative model of *CHATogether* allows collaboration with people who share similar interests, goals, and vision, all of which create a sense of belonging and empowerment. Some participants identified how members support each other’s projects and progress as healing and therapeutic:

I think that all of our healing is tied together in some ways. I think that the integration, healing, growth that I've experienced, I also longed for that and other people, and other people's growth and perspectives and healing also influences and contributes to my growth.

#### Safe space for stigmatized conversations

*CHATogether* provides a place to openly express ideas and thoughts, as well as a platform to try various methods of artistic expression through which to communicate their thoughts and experiences. One participant described the sense of artistic community that *CHATogether* created:

The fact that *CHATogether* is the forum where people connect and talk about these [stigmatized] issues is what I see the most value in. There are not many safe spaces in which you can talk about your feelings, how you feel your racial identity and background really form the way that you experience life is important.

*CHATogether* facilitated conversations about racism, stigma against mental health, and family conflicts. Providing a relatable medium can present a sensitive topic to the audience in a less confrontational way and help initiate difficult but much needed conversations. Participants identified this as a starting point for de-stigmatization. As one participant described, “We come up with a story, a vignette, comics, a conversation that everyone on social media that can help talk about these [stigmatized] topics. We're supporting each other to talk.”

## Discussion

During its first 3 years, *CHATogether* has been a way to join members of the AAPI diaspora across cities, countries, and continents. New members have brought in their unique talents and experiences to build a supportive space for expression without judgment, as well as to grow through mentorship and camaraderie. The COVID-19 and racism pandemics have motivated program members to reach out, connect, and become leaders in personally fulfilling ways. It is the intangible impact of our work that continues to drive *CHATogether* forward.

### Impact on the wellbeing of individuals

Trapped in a foreign country and desperately searching for signs of family’s safety were what numerous international students in US felt in the new year of 2020. They suffered with survivors’ guilt thousands of miles away from loved ones and had no one to turn to. Their challenges resonated with our results about uncertainty and powerlessness during the first years of quarantine. In front of students from both New Haven and Wuhan, our theater skits concretized the core messages of “Do not worry alone” between two friends, and the Wuhan healthcare providers’ internal struggles of away from families while the mere rewards were having the glimmer of hope through connections with patients’ eyes under the personal protective equipment. Audience members uncontrollably sobbed throughout the skit, recognizing themselves and their families in the liminal stage of the play. It is these meaningful projects on *CHATogether*’s digital platform helped unite AAPI youth and enlightened a sense of purpose through productivity and creativity as part of a virtual community without borders. Projects helped transform members’ vulnerabilities into opportunities to pursue excellence and to contributions through performance, use of their talents and creativity, conference presentations, and scholarly publications.

For those identifying with more than one culture, the loss of belonging and conflicting identities as neither Asian nor American could become a significant burden on one’s well-being. Each *CHATogether* component is composed of teams with participants of varying ages, disciplines, and training backgrounds. For example, a high school student member could seek advice from a medical student, and members constantly share ideas, academic career advice, or ideas for cultural peer support, particularly during critical times of anti-Asian hate. This mentorship system provides an upward mobility for AAPI youth and young adults to solidify their heritage, identity, and sense of self. *CHATogether* further enhances AAPI wellbeing through collaborative research projects and mentorship as productive coping strategies against stressors.

### Impact on families

Our results suggest that emotional avoidance that is common in the AAPI community was a contributor to the escalation of conflicts during the pandemic. For a parent growing up in traditional Asian cultures, pressures such as filial piety, collectivism, and selfless conformity to the family unit [44, 45], often make it challenging to empathize with their children. Parents may also struggle in these immigrant children-parent dyads in trying to navigate intergenerational cultural dissonance (ICD), the difference in how immigrant children are better able to adapt to their host country’s culture than their parents. ICD often contributes to family conflict, miscommunication, and real or perceived differences in cultural identity [46, 47]. Increased levels of ICD have been associated with a wide range of adverse outcomes in AAPI families, such as increased parent–child conflict, weaker parent–child bonds, increased risk for youth depression, suicide, social anxiety, decreased academic performance, increased risk for substance use, and increased risk of externalization and subsequent problematic behaviors [46, 48–55]. *CHATogether*’s interactive theater and mental health education arms both center around family conflict inextricably linked to ICD. Instead of avoiding the conflict, our skits illustrate various ways to restore family harmony through mutual compassion and reflection in the child-parent dyad. One of our AAPI parents described her experience with *CHATogether*’s skit as a “Christmas Carol” epiphany. The skit stirred up this parent’s traumatic childhood memories which she never had the opportunity to talk about, which may have influenced her parenting style. She was inspired to share her vulnerabilities publicly and become a role-model for her children to be courageous and open to talk about one’s struggles. Overall, *CHATogether* creates a safe space for stigmatized conversations that would not otherwise be able to be initiated in traditional AAPI families.

### Impact on communities

A few members of *CHATogether* created skits relating to LGBTQIA+ issues uniquely pertinent to the AAPI community. This is one of many AAPI “sub-communities” lacking resources for emotional support. For example, discussions around sexuality, coming out, or talking about gender fluidity to conservative parents are at times unimageable. Cultural taboos, ridicule witnessed over time, a fear of shaming the family or community, or being outright ostracized or disowned were some of the barriers stopping many from opening up to their loved ones [15]. Through writing, creating scripts, and acting, members found a safe space to express themselves without being rejected. Even as some considered the skits to be unrealistically ideal, many found hope and perhaps a means to move the needle on such a challenging societal issue in the AAPI community.

The polarization of racial events and anti-Asian brutality during the COVID-19 pandemic introduced substantial fear and rage. Racial discrimination has been associated with long-term negative consequences [56], including on mental health [57], physical health [58], and even impacting next generations through epigenetic means [59]. As such, there is a need for cultural literacy, anti-stigma campaigns, and mental health prevention. *CHATogether* addresses these needs through community-based outreach and peer support for youths and families. For example, one of our skit conferences featured a conversation between a teen and their parent discussing how AAPI members may support and synergize the *Black Lives Matter* and *Stop Asian Hate* movements. For many AAPIs, discussion of race is often a taboo topic that is rife with conflict and avoidance. *CHATogether* can model non-judgmental conversations by helping performers see each other’s standpoints and understand the impact of racism on mental wellbeing. Dramatized skits also offer “permission to feel” [60] from the collective trauma shared among the attendees. The open discussion fostered by the program helps participants and audience members reflect, engage, and contribute to personal and communal healing and growth.

### Limitations, challenges, and next steps

We concede several limitations to our study: (1) we used convenience, rather than intentional or theoretical sampling. As such, our findings may not reflect the views of those participants or audience members who did not find *CHATogether* to be helpful, or who could have provided constructive feedback to improve the program; (2) many of our study participants were highly motivated and engaged “pioneers” of the program, such that their views may not extend to next-generation colleagues; (3) we did not conduct individual interviews, which could have uncovered more information—particularly dissenting views. In addition to a social desirability bias inherent to a focus group setting [61, 62], a power differential needs to be considered: interviews conducted by a neutral third party would have enhanced this aspect of the study; (4) we did not include audience members or potential stakeholders, such as leaders of AAPI communities; and (5) we recognize the broad umbrella term of the AAPI designation, and recognize that our findings are not intended to generalize across as vast a constituency of unique individuals.

Moreover, we recognize two significant challenges as *CHATogether* moves ahead. Foremost is program sustainability: all program members were recruited on a voluntary basis, and unpaid positions are inherently at odds with long-term planning. Program reproducibility and exportability is a second challenge, even if virtual delivery has already shown promise in uniting motivated members across locations in the U.S. and overseas.

We are optimistic about the program’s future: some participating members have already expressed interest in developing local chapters in their own communities—a standardized curriculum is the logical next step towards this goal. Surveys from community members and event participants, as well as viewership and comments on social media will be useful towards increasing the program’s scalability. In addition, quantitative studies will be helpful in assessing outcomes and refining the program over time. In a mixed methods approach, such studies could be enriched by qualitative components that addressed some of the limitations outlined above.

## Conclusion

Children’s mental health is a national emergency during the COVID-19 pandemic. Witnessing the rise of health disparities in minority communities while the current healthcare system is falling short to meet the needs of communities is alarming. *CHATogether* may serve as a model for a novel practice for AAPI mental health by (1) introducing storytelling to capture relatable narratives around culture and family; (2) delivering community strengths-based recovery through harnessing the talents of youths, young adults, and parents; (3) providing digital resources and peer support to address collective trauma from racism and microaggressions; and (4) promoting a child-parent prevention intervention to embrace a cultural sense of self, purpose, and altruism in unprecedented times.

## Data Availability

The datasets obtained and analyzed during the current study are available from the corresponding author on reasonable request.

## Appendix

**Table:** *CHATogether* study participant demographics (n = 20)

Characteristic	n
Involvement as program leaders	
Since program inception (3 years)	7
< 6 months	6
Non-member volunteers^a^	6
Self-described AAPI ancestry/affiliation^b^ (-American)	
Chinese	9
Taiwanese	4
Hong Kong	3
Korean	3
Malaysian	3
Vietnam	1
Non-AAPI	5
Self-described AAPI origin	
Predominant	12
Partial	8
Acculturation process (years since arrival in the US, or description)	
< 5	4
5–10	4
> 10	3
Second generation living with immigrant parents	9

^a^Non-members were randomly recruited through a word of mouth, advertisements, or event participants who expressed interest in the program

^b^Total is more than 20, as several participants identified with more than one category
